# Oral and Topical Probiotics and Postbiotics in Skincare and Dermatological Therapy: A Concise Review

**DOI:** 10.3390/microorganisms11061420

**Published:** 2023-05-27

**Authors:** Carolina Vieira De Almeida, Emiliano Antiga, Matteo Lulli

**Affiliations:** 1 Beauteesum Research and Innovation, 25122 Brescia, Italy; 2 Department of Health Sciences, Section of Dermatology, University of Florence, 50139 Florence, Italy; emiliano.antiga@unifi.it; 3 Department of Experimental and Clinical Biomedical Sciences “Mario Serio”, University of Florence, 50134 Florence, Italy; matteo.lulli@unifi.it

**Keywords:** oral, postbiotics, probiotics, skin, skincare, topical

## Abstract

The skin microbiota is a pivotal contributor to the maintenance of skin homeostasis by protecting it from harmful pathogens and regulating the immune system. An imbalance in the skin microbiota can lead to pathological conditions such as eczema, psoriasis, and acne. The balance of the skin microbiota components can be disrupted by different elements and dynamics such as changes in pH levels, exposure to environmental toxins, and the use of certain skincare products. Some research suggests that certain probiotic strains and their metabolites (postbiotics) may provide benefits such as improving the skin barrier function, reducing inflammation, and improving the appearance of acne-prone or eczema-prone skin. Consequently, in recent years probiotics and postbiotics have become a popular ingredient in skincare products. Moreover, it was demonstrated that skin health can be influenced by the skin–gut axis, and imbalances in the gut microbiome caused by poor diet, stress, or the use of antibiotics can lead to skin conditions. In this way, products that improve gut microbiota balance have been gaining attention from cosmetic and pharmaceutical companies. The present review will focus on the crosstalk between the SM and the host, and its effects on health and diseases.

## 1. Introduction

The last decade has seen an explosion in microbiota research, which has enabled a better understanding of its structure and function, leading to potential opportunities to develop next-generation microbiome-based drugs and diagnostic biomarkers. These studies have demonstrated that trillions of microbes live within our bodies in a deeply symbiotic relationship. They have provided evidence that microbial populations vary across body sites, driven by differences in the environment, immunological factors, and interactions between microbial species [1]. To better understand the function of the microbiome, it is fundamental to consider how microbes that live on body superficies can influence systemic performances.

The skin is the primary physical barrier that protects our bodies from potential invasion by pathogens or toxic substances, but skin does not act alone: microorganisms that live in symbiosis with us and occupy a broad array of skin niches help to protect our body against harmful situations. Microorganisms from the skin can act directly to defend the host against pathogens, control inflammation, and modulate the adaptive immune pathways [2,3,4].

It is important to highlight that the use of probiotics and postbiotics in skincare and dermatological conditions is in its early stages, and its applications require more extensive human trials to verify the potential risks and reliability, as well as the precise direct and indirect actions of them. In the present review, we grouped the most relevant information about skin microbiota composition, distribution, role in host’s health and the potential use of topical and oral pro- and postbiotics to regulate it. In Table 1, we define the main terms that we use in this article to avoid possible doubts and confused interpretations.

## 2. The Skin

**Figure 1:**
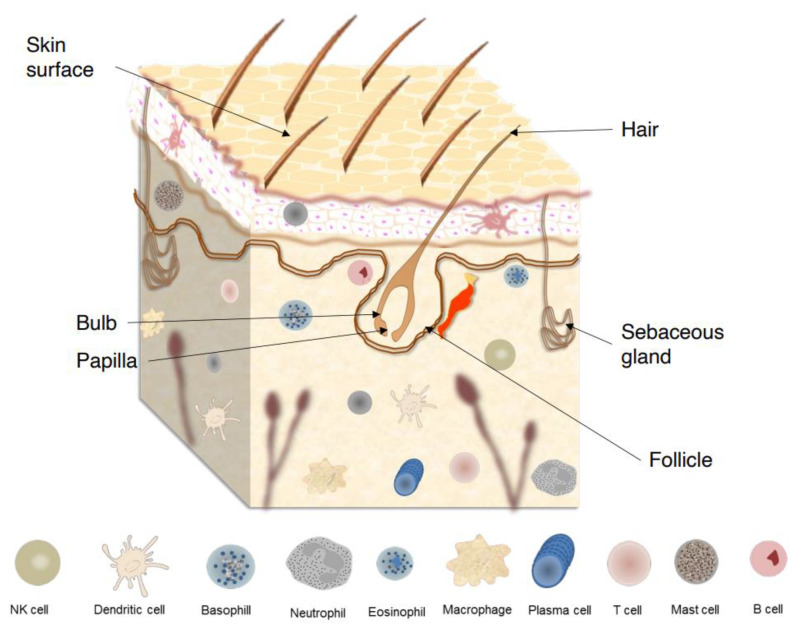
Skin surface, skin appendages, and major components of skin immune system.

The human skin comprises a variety of unique and uneven regions, with lines, ridges, and invaginations from skin appendages unevenly distributed over its surface (Figure 1). The outermost layer of epidermis is the stratum corneum (SC), which consists of piles of dead keratinocytes (corneocytes) and intercellular lipids. Due to a specific and unique type of functional cell death named corneoptosis, keratinocytes are converted into corneocytes, which remain functional in the SC and act as a functional barriers. This barriers protect the body against mechanical stresses, dehydration, toxic substances, and pathogen invasion, enabling terrestrial vertebrates succeed survive in nonaquatic environments [5,6].

The skin, also known as ‘acid mantle’ [7], consists of a lipid- and protein-laden cornified layer dotted with hair follicles and glands that secrete lipids, antimicrobial peptides (AMPs), enzymes, salts, and many other compounds. Over the SC, the pH is gradual, but in general, the natural skin surface, when in good conditions, presents an average pH below 5.0. This acidity is essential to maintain the balance of skin microbiota, as well as to support important physiological processes, such as the formation of the lipid barrier and SC homeostasis. In addition, the skin surface is a high salt, desiccated, and aerobic environment [2,8,9]. These odd characteristics of the skin surface make the skin physically and chemically distinct from another microbe-rich barrier sites such as the small and large intestines which are characterized by a polysaccharide-rich [10] and neutral pH surface [11].

Because it is always exposed to possible pathogens and is subject to sterile inflammation, comprising tumor immunity, allergy, and autoimmune responses, an appropriate functioning of skin defense based on complex action of a variety of complementary systems is fundamental. Complementary to the physical barrier is an active synthesis of gene-encoded host defense molecules such as proteases, lysozymes, antimicrobial peptides (AMPs), cytokines, and chemokines that serve as activators of the cellular and adaptive immune responses [2].

Maintenance of skin homeostasis upon inflammatory challenges requires various types of immune cells that are resident in skin or are recruited (Figure 1). For instance, in the epidermis, keratinocytes produce AMPs that exhibit direct bacteriostatic or bactericidal activity, promoting the recruitment of immune cells such as Langerhans cells (LCs) [4]. These cells are a subset of tissue-resident macrophages that reside between keratinocytes and, upon further differentiation, acquire dendritic cell (DC)-like phenotype and functions [12]. On the other hand, in the dermis, several types of innate immune cells, including dermal DCs, macrophages, mast cells, γδ T cells, and innate lymphoid cells (ILCs) are found [13]. Additionally, it is well established that both innate and adaptive immunity processes of the skin are profoundly influenced by different microorganisms’ species athwart their metabolites and/or structural components [14]. Symbiotic microorganisms protect against invasion by more pathogenic or harmful organisms, as they are able to modulate skin immunology, maintain skin homeostasis, stimulate and activate various immune responses, metabolize natural products, and produce AMPs, influencing the natural course of several skin diseases [15,16]. However, since the corneocytes from SC are dead cells, they do not express active biological sensors for microorganisms; thus, the greater influence of the microorganisms on cutaneous immunity occurs in the skin appendages.

The irregular topography of the skin, and its different site characteristics and specialized niches, permits it to provide distinctive habitats for microorganisms, making it an ecosystem with perfect growth conditions for both resident (symbiotic) and transient (opportunist pathogenic) microbiota [17,18]. The skin microbiota is composed of a diverse collection of microorganisms, including bacteria, fungi, viruses, and mites [19] (principally Demodex mites) [20]. Its composition is supposed to be relatively stable, with a heterogeneous distribution of its major microenvironments which present different physiological compositions: (1) sebaceous (face, chest, and back), (2) moist (elbow, knees, genitalia), (3) dry (palms), and (4) foot-specific [14,21,22]. Moreover, distinct factors can interfere with the composition of the skin microbiota. These factors include gender [23,24], ethnicity [25], age [26,27], diet, lifestyle, environment (external factors), previous antibiotic treatment, clothing type, skincare routine, and hygiene frequency [28,29].

Microorganisms, however, occupy a wide range of skin niches and in addition to the external interfollicular epithelial surface, they can also be found on the skin appendage surface and below the basement membrane [30,31]. An abundant number of bacteria reside within the hair follicle even if these structures are not directly exposed to the general external environment [32]. Nevertheless, differently from skin surface, the invaginations that form follicle-sebaceous units are comparatively anaerobic and more lipid-rich since they are colonized by a different microorganism composition [33].

## 3. The Skin Microbiota

The skin ecosystems are composed of diverse microorganisms that interact with the human body, including host epithelial and immune cells, as well as with other microorganisms sharing the same niche [34]. To establish the microbiota, the skin provides essential nutrients, such as amino acids from the hydrolysis of proteins, fatty acids from the stratum corneum, sweat, lipid hydrolysis or sebum, and lactic acids from sweat [35]. Since this symbiotic relationship between the host and commensals is crucial for several physiological processes, commensal-specific T cells distinguish resident microorganisms from pathogens to promote commensal tolerance [36,37].

Microbiota colonization begins at birth and its composition is influenced by several intrinsic (skin site, age, ethnicity, gender, intra- and interpersonal variability) and extrinsic (lifestyle, cosmetic use, use of antibiotics, hygiene routine, climate, seasonality, and geographical location) factors [38]. During puberty, it is possible to observe a decrease in the abundance of Firmicutes, including *Staphylococcus* and *Streptococcus* species, and increased predominance of *Corynebacterium* and *Cutibacterium* (previously known as *Propionibacterium*). On the other hand, the adult microbial composition remains constant over time despite its continuous exposure to the environment [39,40].

The skin microbiota composition is distinctive to each person as well as to each part of the body; however, *Cutibacterium, Corynebacterium,* and *Staphylococcus* represent the three most dominant microorganism genera in the human skin at the genus level [41]. Viruses have been less investigated but can be also found on skin surface in scarce concentrations, such as the ß and γ human papillomaviruses, which are the most common components of skin microbiota [42].

The bacterial diversity is heterogeneous across skin regions due to the variety of glands and density of hair follicles, which create complex and distinct physical and chemical niches for microbial growth. Sebaceous sites, for example, have the lipophilic *C. acnes* and other *Cutibacterium* as the dominant species. Over moist sites, instead, *Staphylococcus* and *Corynebacterium* are primarily the first colonizing genera, while *Lactobacillus* is predominant across the female genital tract [14,43,44]. Fungal communities are usually seen in great abundance on foot sites. Within other species, the most abundant are *Aspergillus, Malassezia, Rhodotorula, Cryptococcus,* and *Epicoccum*. *Malassezia* is the most abundant species on core-body and arm sites [45].

Facial skin microbiota is particularly studied due to its role in aging, acne, and rosacea. It is composed mainly by *Proteobacteria* (32.91%), *Firmicutes* (28.69%), *Actinobacteria* (33.07%), and *Bacteroidetes* (3.08%), with significant age-dependent profile variations. Subjects aged 36–52 years old have the highest Shannon index, which gives an estimate of the species diversity within a community, as recently demonstrated [46]. The same authors also demonstrated that, in women aged 53–68, the class of *Actinobacteria*, the order of *Corynebacteriales*, the family of *Nocardioidaceae*, and the genus of *Lactococcus* were significantly more abundant [46]. These data are in line with that obtained in other studies about the skin pH variation in different periods of life, which demonstrate that skin pH values are higher at young (newborns) and older (70–80 years) ages [47,48,49]. Microbiota profile also changes according to the facial area: while highest alpha-diversity in richness and evenness scores is observed on cheek sites, forehead sites present the lowest index of diversity [50].

## 4. Skin Dysbiosis and Cutaneous Alterations

How homeostasis is maintained and shaped by the skin microbiota is still not clear; however, it is well established that the balance between members of skin microbial communities plays a pivotal role in guarding against cutaneous disorders. Changes in composition and the lack of balance among microbial communities is referred to as dysbiosis, a condition that may lead to the onset or progression of diseases. Pronounced dysbiosis on skin may result in several cutaneous diseases including atopic dermatitis (AD), seborrheic dermatitis (SD), rosacea, alopecia areata (AA), and acne [14,51,52].

AD affects 15–20% of children and 2–10% of adults, and results from a complex interaction between genetic susceptibility, barrier dysfunction, innate and adaptive immunity, and microbiota [53,54]. When compared to healthy controls, AD patients present a loss of diversity with increased abundance of *S. aureus* and depletion of *S. epidermidis* and *Corynebacterium* spp. [55,56]. Disease severity is also associated with decreased gut microbiome diversity [57,58].

A dysbiotic scenario with significant alterations of bacteria populations on the scalp skin of SD patients, another common dermatological disorder, was also demonstrated. SD symptoms include skin erythema, flaking, and pruritus. Patients with SD show reduction of *Corynebacterium* spp. and domination at taxa level by Firmicutes, while at genus level by *Pseudomonas* spp., *Staphylococcus* spp., and *Micrococcus* spp. [59].

It is also suggested that, in an undefined subset of predisposed rosacea patients, although microorganisms may not be central causative factors, skin dysbiosis may act as a potentiator of inflammation or trigger factors. Many systemic comorbidities are associated with this chronic inflammatory cutaneous disorder, such as neurological, psychiatric, gastrointestinal, cardiovascular, and autoimmune diseases [60]. In recent years, it was demonstrated that the commensal *Demodex folliculorum* could participate in the pathogenesis of rosacea, due to the fact that it acts as a vector of endosymbionts. Until now, different endosymbiotic microorganisms were isolated from *D. folliculorum*, such as *Staphylococcus epidermidis*, *Cutibacterium acnes,* and some species of *Bacillus,* such as *B. oleronius*, *B. cereus*, *B. pumilus*, and *B. simplex* [61,62,63,64].

The role of the skin microbiota on AA is probably associated with its specific microbial composition, mainly on the proximity of the bulge (stem cell niche) and the bulb (cellular division site to build a new hair) of hair follicles (HF). Variations in its balance observed in AA patients are being related to modulation of immune function, loss of homeostasis, and intense peribulbar inflammation [65]. It is important to notice that in addition to AD, the gut microbiome has been related to autoimmunity in AA [38].

The phylogroup diversity loss was also been associated with acne by several authors. Despite years of being largely associated with *C. acnes* proliferation, nowadays loss of diversity is being considered the main trigger for acne, since there is no significant difference in the concentration and bacterial load of *C. acnes* among acne patients and healthy subjects (reviewed by Carmona-Cruz et al.) [38]. Finally, it is important to emphasize the essential symbiotic relationship between the skin microbiota and skin physical, chemical, and immunologic barriers [66]. Since skin microbiota is crucial for maintaining the integrity of the epithelial barrier, the overuse of antibiotics can change its composition, eliciting critical skin changes such as cutaneous infections and inflammatory disorders [67].

## 5. Skincare and Skin Microbiota

Skin aging is a complex biological process influenced by the combination of endogenous (intrinsic) and exogenous (extrinsic) factors. The main intrinsic factors are genetics, cellular metabolism, hormones, and metabolic processes, while the main extrinsic factors are chronic light exposure, pollution, ionizing radiation, chemicals, and toxins [68].

Skin suffers progressive morphologic and physiologic decrement with increasing age and provides the first obvious evidence of the aging process. A daily skincare routine is essential to enhance smoothness, regenerate it, give strength and elasticity, as well as to prevent the degradation of collagen and elastin, which reduce the formation of wrinkles [69,70]. Moreover, various studies have examined whether cosmetics could affect skin microbiota composition and balance and have demonstrated that certain bacteria can grow by metabolizing some cosmetic ingredients [71,72]. It has been demonstrated that even with regular showering, many molecules from personal skincare and hygiene products can last on the skin for weeks after their first use. In this way, a single application of these products may alter the skin chemistry and microbiota for long periods [71].

After analyzing the effects of daily use of skincare products, a recent study has suggested that these products might improve skin biophysical parameters such as smoothness and hydration level, while pH and sebum content were maintained. The authors also suggested that a daily skincare routine might improve the microbial health of facial skin and increase the Shannon diversity [73]. As is known, high alpha diversity is considered a hallmark of healthy skin microbiota and a balanced skin microbiota is known to play an important role in skin health, as any alterations lead to the overgrowth of pathogenic strains linked to various skin diseases [74]. It was also suggested that skincare products favor the growth of *Cutibacterium* and *Staphylococcus* [73] strains that can uphold the microbial equilibrium by inhibiting pathogen growth [51] and stimulating human sebocytes and keratinocytes to produce AMPs [75]. These results suggest that appropriate use of skincare products might preserve skin health.

Another study revealed that skin metabolome and microbiome can be altered with changes in the hygiene routine, but that this alteration has responses that are specific to the individual [71]. However, as previously cited, the better conditions of skin biophysical parameters, such as barrier function, scaling, and moisturization, are found when its pH average is below 5. Use of products with high pH alkalizes the surface of the skin, which can cause irritability and an increase in dehydration, as well as changes in the microbiota composition by promoting their dispersal from the skin [9,76]. Therefore, the pH factor should be given due consideration by consumers and cosmetics producers, especially when it comes to sensitive and acne-prone skin. In fact, in recent years, several cosmetic brands have started to regard the impact of their products on skin microbiota, causing a new branch of the market to flourish, i.e., R&D startups that offer “Microbiome-friendly” certification to final cosmetic products. These certifications aim to validate not only that the product is contamination-free, but also that microbiome diversity will be preserved, that specific bacteria of the targeted area will remain unharmed, and that skin balance will not be disturbed.

It is fundamental to consider, however, that physiologic and molecular responses of the skin to environmental factors depend on the skin type, which means that every individual presents different demands to prevent skin damage and improve regeneration. This opens a promising new commercial approach of personalized skincare, an actively growing area of a new generation of skin products based on the individual profile.

## 6. Topical Use of Probiotics

The skin microbiota, its interactions with the environment, and the possibility of manipulating it to address cutaneous conditions have opened exciting new paths for dermatological therapies. Therefore, the cosmetic and pharmaceutical industries have been engaged in ordering solutions coming from nature, especially probiotics and postbiotics.

The use of probiotics as a potential alternative to antibiotics was previously demonstrated in the treatment of inflammatory bowel disease (IBD) [77] and several atopic conditions [78,79]. Topical probiotics were first proposed as a treatment for cutaneous diseases in 1912 to treat acne and seborrhea [80] that involves the transfer of laboratory cultured live bacteria in a dose suitable for skin, to equilibrate the skin microbiota, reestablishing the immune homeostasis [81]. Such products rely on the fact that skin immune setting is highly dynamic and can be rapidly remodeled by encounters with specific commensals. In this way, through essential interactions, topical probiotics implement both the establishment and restoration of cutaneous homeostasis [82].

It is known that, under specific conditions, probiotics can persist and successfully colonize the skin [83], inducing keratinocytes and sebocytes to produce AMPs or other metabolites that can directly inhibit or kill pathogenic microorganisms, shaping microbial communities [84,85,86], and establishing a synergistic effect which improve the ecology of skin microbial communities [87]. Considering that the first step in bacterial colonization that leads to infection is cell adhesion, the ability of probiotics to invade and adhere to keratinocytes, promoting inhibition of pathogen binding to these cells, reinforces that probiotics could be used to reduce adhesion of some pathogens to skin [88]. Moreover, the potential antimicrobial use of AMPs produced by probiotics has been shown. Indeed, it has been observed that some coagulase-negative *Staphylococcus* (CoNS) species, including *S. hominis* and *S. epidermidis,* can produce commensal-derived AMPs (phenol-soluble modulins and *Sh*-lantibiotics), which exert selective antimicrobial activity and cooperate synergistically with host-derived AMPs, such as cathelicidin LL-37, to inhibit survival of skin pathogens [84,89,90,91]. It has been proposed that by directly producing AMPs, the microbiome provides the first line of defense against microbial pathogens, synergizing with the host innate immune response as a second potent line of defense [84]. This normal microbial defense strategy against colonization and transmission of bacterial pathogens should be exploited for anti-infective therapeutics [89,90,91].

Studies on animals and humans have demonstrated that different probiotic bacteria strains, mainly when associated with konjac glucomannan hydrolysate (GMH) prebiotics, are able to significantly inhibit the growth of skin bacterium species associated with acne, such as *C. acnes* [92]. Moreover, the activities of several cytokines, inflammatory mediators, and related signaling pathways can be inhibited by some probiotics such as *L. plantarum* and *L. acidophilus*. Specifically, *L. plantarum* decreases lipid production, which is further attenuated in the presence of pro-inflammatory mediators in a 2D human primary sebocyte culture as a model for treatment for mild-to-moderate papulopustular acne. A significant decrease in the production of inflammatory mediators (IL-1, IL-6, and IL-8) in the presence of LPS, when compared to non-treated human primary sebocytes, was also observed, together with a mean reduction of 62% of the overrepresented pathogens causing acne, including class-A *C. acnes* and *S. epidermidis* [93]. *L. plantarum* was also demonstrated to be effective against five skin pathogenic strains related to skin diseases [94], as well as to exert anti-aging effects, by significantly increasing dermal density and better barrier function [95], suggesting that it could be administered topically or orally as alternative therapeutics for skin infections.

Additionally, some lactic acid bacteria such as *Streptococcus thermophiles* enhance ceramide production both in vitro and in vivo, when topically applied as a cream. Ceramides are well-known for improving acne by confining water in the skin and by their antimicrobial activity against *C. acnes*, helping strengthen the skin barrier and soothe the irritated skin, which is beneficial to acne-affected skin.

Another potential use of topical probiotics is the prevention and treatment of skin photoaging, which is closely associated with the reduction in MMP synthesis and collagen production, increase of ROS-induced damage, and activation of the MAPK and NF-kB signaling pathways. For example, when associated with live *Lactobacillus*, the topical use of *Agastache rugosa*-fermented extract (ARE-F) decreased UVB-induced ROS, MMP-2, and MMP-9 levels while enhancing UVB-induced levels of total glutathione and superoxide dismutase activity in a UV-B-irradiated human keratinocyte cell line (HaCaT) [96]. A second study demonstrated that the topical application of a specific *L. acidophilus* strain can protect epidermal cells against UVB-induced photodamage not only by enhancing the skin hydration factors and the activity of antioxidant enzymes, but also by suppressing the MMP levels through inhibition of the MAPK signaling pathway [97].

Regarding the effects of probiotics on the wound healing process, studies in animal models with acute uninfected wounds, thermal wounds, and diabetic ulcers have demonstrated contradictory results. Some studies reported a positive curative effect of probiotics by increasing granulation tissue deposition, improving collagen concentration, and stimulating angiogenesis, while in some others the wound healing process did not improve. It has been demonstrated that the topical use of *Nitrosomonas eutropha,* for example, reduces the number of pathogenic bacteria on the skin and improves skin healing, wrinkle depth and severity [98]. Conversely, in experiments by Twetman et al., the wound healing process was not influenced by *L. reuteri* supplements [99]. However, it is important to highlight that no adverse effects, such as increase in the healing time of wounds, were observed when probiotic therapy was used in either of these studies.

Despite several health benefits, some limitations on the safety of the use of probiotics may present, mainly in people with weak immune systems, such as infants, expectant women, and elderly individuals [100,101]. Further studies are necessary to demonstrate efficacy, the mechanisms of action, and mainly the safety of topical use of probiotics as dermatological therapy and skincare since research is still in the initial stages. The most potential side effects include antibiotic resistance transfer among pathogens, allergic reactions, and bacteremia [102].

Moreover, due to their sensitivity to temperature, humidity, and air conditioning, several factors can affect probiotics quality during storage or delivery [103]. To address these concerns, some alternatives have been recently suggested such as prebiotics, synbiotics, paraprobiotics, and postbiotics.

## 7. Topical Use Postbiotics

Dead cells present on probiotics are able to produce biological responses as effectively as their live equivalents. In fact, it was demonstrated that in adequate amounts, when administered orally or topically, dead microorganisms confer a benefit on the human or animal consume [104,105]. However, since they cannot be classified as probiotics, for dead microorganism products new terms have been coined such as postbiotics, paraprobiotics, non-viable probiotics, inactivated probiotics, tyndallized probiotics, or ghost probiotics [106,107,108]. The inactivation of viable cells could be performed by using various mechanical methods, such as sonication, thermal treatment, electromagnetic radiation, high pressure, or ultraviolet irradiation [109].

The term postbiotic was recently defined by the International Scientific Association of Probiotics and Prebiotics as “a preparation of inanimate microorganisms and/or their components that confers a health benefit on the host” [110]; thus, they cannot colonize the host. Other components that are not required in a postbiotic, such as physiologically active microbial cellular components (cell wall fragments or enzymes) or functional bioactive substances (products or metabolic byproducts) such as vitamins, short-chain fatty acids (SCFAs), proteins and antibiotics, can contribute substantively to the complexity and functionality of the postbiotic preparation [111]. It is important to highlight that purified metabolites and purified cell components have specific chemical names of their own that can be used; thus, they do not fall under the scope of postbiotics, and should be referred to as microbe-derived substances.

**Figure 2:**
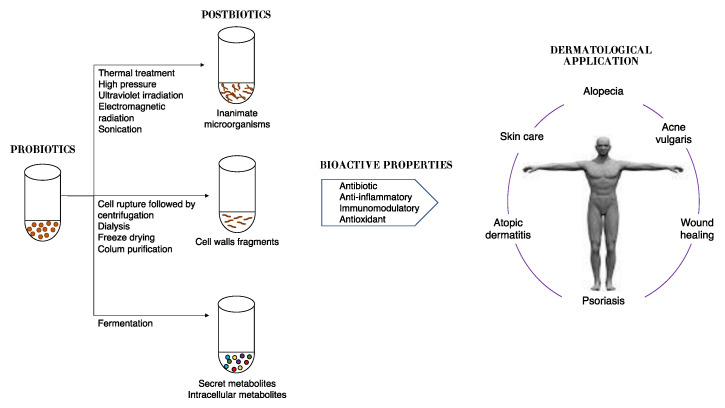
Schematic representation of approaches in postbiotic production and their benefits to skin health.

Therefore, postbiotics can be defined as cell-free supernatants without metabolite specification/individualization, cell wall components and/or intracellular compounds (Figure 2). These compounds have many potential health benefits including anti-inflammatory, antioxidant, immunomodulatory, antimicrobial, and anti-ageing/anti-senescence activities (reviewed by Duarte et al., 2022) [112]. There are several advantages to postbiotics over probiotics, such as defined chemical composition, no ability to transfer antibiotic resistance—which permits their use in immunosuppressed people—as well as their stability over a wide temperature and pH range and longer shelf life [113]. Moreover, since there is no need to maintain viable cells in formulations with postbiotics, they are an innovative cosmetic ingredient [114].

Though evidence of microbiota modulation by postbiotics in humans is limited, due to the benefits overcited, the term postbiotic is emerging on commercial products for humans and animals [105]. Moreover, great effects on the skin have been seen, such as improved skin moisturizing [115], prevention of wrinkle formation [116], and amelioration of atopic dermatitis [117,118].

The topical application of postbiotics from *L. fermentum*, *L. reuteri,* and *Bacillus subtilis natto*, when associated with a cold cream, for example, is being considered as a novel therapeutic approach to accelerate the wound healing process, with an earlier complete epithelization and absence of skin inflammation in the group treated with *L. reuteri* [119]. In vitro antioxidant assays showed significant free radical scavenging activity of a cell-free, casein-free supernatant of *L. helveticus* strain fermented milk against UVB-induced skin photodamage. The melanin production by melanocytes cells in vitro and the expression of proteins needed for melanin synthesis were inhibited by supernatant. In addition, reduction of transepidermal water loss, increase of epidermal thickness, and favorably modulated lipid peroxidation levels were observed [120]. A blend resulting from the co-fermentation of three proprietary probiotic strains, *L. plantarum* (AN057), *L. casei* (AN177), and *S. thermophilus* (AN157), showed beneficial effects on pore size and wrinkle depth reduction, as well as skin moisture and elasticity increase [121].

The topical use of *S. thermophiles* lysate containing sphingomyelinase, an enzyme that converts sphingomyelin to phosphocholine and ceramide, demonstrated a significant increase in stratum corneum ceramide levels, improved the lipid barrier and reduced water loss [122]. A postbiotic from *L. plantarum* K8 strain lysate was recently suggested as a functional ingredient for moisturizing products, since it was able to increase the mRNA expression levels of moisturizing factors, including HAS2 and AQP3 [123]. *L. plantarum* ferment lysate was also demonstrated to significantly improve acne lesions, with significant decrease of transepidermal water loss and sebum production after 4 weeks of treatment [124].

Since the lysate from *Vitreoscilla filiformis* can bind to toll-like receptors present on different skin cell types, it can display multiple beneficial effects on skin health. It can modulate the skin immunity by decreasing skin inflammation, stimulating skin defenses, and improving the skin barrier by enhancing tight skin junctions [125]. The pure extract of *V. filiformis* lysate, Vfe, can regulate skin homeostasis, predominantly by inducing the release of IL-10 through the TLR2 pathway in dendritic cells, inducing T regulatory cell functions, as well as significantly stimulating the chemotactic migration of polymorphonuclear neutrophils [126] and the expression of TNFAIP3/A20 in human epidermal keratinocytes, indicating an anti-inflammatory activity [127].

The LactoSporin^®^ formulation, a cream containing cell-free supernatants of *Bacillus coagulans* and inactivated cells of *Bacillus longum*, demonstrated effectiveness in mild-to-moderate acne lesions and other seborrheic conditions in both male and female subjects, due to its ability to reduce sebaceous secretion and oily, greasy nature of the skin even better than benzoyl peroxide [128]. Moreover, a recent trial with a skincare cream containing a bacterial lysate of *L. plantarum* VHProbi^®^ V22 isolated from fecal samples of infants reduced the severity of acne in subjects with mild-to-moderate cases [129].

Topical application of a postbiotic developed from *Bifidobacterium lactis* revealed efficiency in improving general aspects of dandruff, possibly by reinforcing skin barrier function and modulating the skin immune system, which leads to preservation of skin homeostasis and rebalancing of the microbiota of the scalp [130]. The use of TR-PRP plus-Celsi, a formulation for topical application containing plantaricin A bioactive peptides (PlnA–small bioactive peptides produced by *Lactobacillus plantarum* strains), postbiotics, and *Lactobacillus kunkeei* isolated from bee bread, and *Tropaeolum majus* flower/leaf/stem extract, resulted in a significant improvement in patients diagnosed with alopecia areata [131].

## 8. Use of Oral Probiotics and Postbiotics for Ameliorating Skin Health

There is a relationship between the gut microbiome and skin health, termed the gut–skin axis. The gut–skin axis results from the resemblance between these organs: both are highly innervated and vascularized, and they are essential for immune and neuroendocrine function [132]. There are some interesting similarities between inner surface of the gut and the surface of the skin: both are covered by epithelial cells, which maintain an important link between the internal body and the external environment, acting as a first line of defense and thus preventing the entry of microorganisms [133]. Moreover, gut and skin tissues are the two major niches that host prokaryotic and eukaryotic symbiotic microorganisms due to their high cellular turnover rate, which determines a low adherence and infection by the colonizing microbiome [34,134,135].

Inflammatory skin diseases can be associated with disruptions of gut microbiome mediated by metabolites released by the microorganisms, as well as increased inflammatory mediators [136,137]. There is growing evidence supporting that intestinal dysbiosis is observed in common inflammatory skin diseases such as AD, psoriasis, rosacea, and acne vulgaris (reviewed by Szántó et al.) [137], which increases the potential of oral probiotics as a treatment option for skin disorders.

Oral probiotics are a group of living microorganisms that could change the gut microbiota and induce a protective effect on specific skin cells by inducing a series of immune and inflammatory responses. In recent years, several studies have indicated that the use of oral probiotics is beneficial to the skin. For example, the use of a probiotic consisting of *Lactobacillus acidophilus*, *Lactobacillus delbrueckii* subspecies *bulgaricus*, and *Bifidobacterium bifidum* was shown to be similarly effective to minocycline 100 mg daily for acne [138]. An important reduction of acne lesions, modulation of the skin biophysical properties and sebum excretion rate were observed with the use of a supplement compose by a mix of different strains from *Bacillus* species [139]. A supplementation with the probiotic strain *Lactobacillus rhamnosus* SP1 (LSP1) in adults with acne normalizes skin expression of genes involved in insulin signaling and improves the appearance of the skin [140].

Skin photoaging is mainly caused by UVR exposition; however, it is also closely associated with changes in metabolic capacity, cofactor and vitamin metabolism, antibiotic biosynthesis, glycolipid metabolism, and fatty acid biosynthesis. A growing amount of evidence associates oral probiotics with skin photoaging control by positively modulating gut–skin microbial interaction, reducing the oxidative stress level, inhibiting the inflammatory cascade, maintaining immune homeostasis, and inhibiting extracellular matrix remodeling [141].

Oral consumption of some postbiotics has been shown to respect microbiota equilibrium and to restore/improve the skin barrier integrity through antioxidant activity and inhibition of enzymes associated with extracellular matrix disintegration. Thus, it is postulated that postbiotics can improve UV protection, delaying the ageing process of skin cells [142]. Intake of heat-killed cells of *L. lactis* strain H61 was demonstrated to improve some skin properties such as suppression of dehydration due to seasonal change, showing significant improvements in elasticity of skin on the inner forearms. However, the effects on some skin properties, such as melanin content in the cheek, were variable according to the age of the patient [143].

Efficacy limitations of topical strategies to treat skin alterations have emerged, thus prompting the evaluation of therapeutic oral probiotics administration as an efficient alternative. Modulating the gut microbiota with symbiotics (probiotics plus prebiotics) or prebiotics also demonstrated great effects on skin health. Oral administration of Lactocare^®^, a symbiotic formulation, when associated with topical administration of hydrocortisone demonstrated improvement in the psoriasis indexes [144]. Mice with AD, when fed with olive-derived antioxidant dietary fiber, had gut microbiota composition modulated, improvement of cytokine profile and butyrate production influencing AD-associated immune response [145].

Decolonization of *S. aureus* within the human body is considered a major goal to prevent or treat its infection, but topical treatments with antibiotics or antiseptics shows limited therapeutic effect. A phase 2 clinical trial recently demonstrated that oral administration of *Bacillus subtilis* succeeded in reducing about 95% of the total number of *S. aureus* colonies in human bodies, not only in the intestine but also in distal sites such as the nose, without a substantial effect on the microbiome [146].

## 9. Conclusions

Skin microbiota is an important target of therapeutic interaction since, more than just constituting a physical barrier to pathogenic bacteria colonization of the skin, it can promote immune system modulation and reduce inflammation, playing an important role in dermatological diseases. Because skincare products can alter molecular and bacterial diversity as well as the dynamics and structure of molecules and bacteria on the skin, several clinical trials are being carried out to study the efficacy and adverse effects of topical pro and postbiotic formulations for the treatment of different skin problems. Moreover, in the same way that modulation of the intestinal microbiome may lead to systemic effects, targeting skin health through modulation of intestinal microbiome with the use of pre- and probiotics seems to be a promising alternative therapy. We believe that further investigations are necessary to better understand how lifestyle characteristics such as diet and exercise, as well as the use of some medications, can shape the skin microbiota and consequently the host health.

## Figures and Tables

**Table 1:** Definition of key terms used in this paper that must be clear to better understand this review.

Expression	Definition
Microorganism	A microorganism, also known as a microbe, is an organism that is microscopic. Microorganisms can be bacteria, fungi, archaea, protists, viruses, and prions.
Microbiota	Usually refers to microorganisms that are found within a specific environment. All the microorganisms found in an environment, including bacteria, viruses, and fungi.
Microbiome	The set of genomes of microorganisms in symbiosis with the human host, including microorganisms (alive or dead) and free DNA, whereas microbiota refers to microorganisms living in or on a defined ecosystem.
Ecosystem	A biological community of interacting organisms and their physical environment.
Probiotic	Live microorganisms that have a positive influence on the host organism due to modulating immune response and competing with pathogenic bacteria.
Prebiotic	Non-digestible food ingredients which selectively stimulate the growth and activity of bacterial species with a positive influence on the health of the host organism.
Postbiotic/ Paraprobiotic	Non-viable probiotic, just its cell components and metabolites with great immunomodulation ability.
Synbiotic	A nutritional supplement comprised of prebiotic and probiotic ingredients, with potential immunomodulating and gastrointestinal (GI) flora-restoring activity.

## Data Availability

Not applicable.
